# Effect of Injectable Acellular Adipose Matrix on Soft Tissue Reconstruction in a Murine Model

**DOI:** 10.1007/s00266-024-03924-3

**Published:** 2024-03-18

**Authors:** Jaewoo Kim, Vinh Vuong The Tran, Ki Yong Hong, Hak Chang

**Affiliations:** 1 Department of Plastic and Reconstructive Surgery, Seoul National University College of Medicine, Seoul, Republic of Korea; 2 Department of Plastic and Reconstructive Surgery, SMG-SNU Boramae Medical Center, Seoul, Republic of Korea; 3 Hi-Tech Center, Vinmec Healthcare System, Hanoi, Vietnam; 4 Department of Plastic and Reconstructive Surgery, Seoul National University Hospital, Seoul National University College of Medicine, 101 Daehak-ro, Jongno-gu 03080 Seoul, Republic of Korea

**Keywords:** Acellular adipose matrix, Decellularized adipose tissue, Fat graft, Volume effect, Injectable

## Abstract

**Background:**

The extracellular matrix isolated from adipose tissue, known as acellular adipose matrix (AAM), represents a novel biomaterial. AAM functions as a scaffold that not only supports stem cell proliferation and differentiation but also induces adipogenesis and angiogenesis. This study aims to investigate the volumetric effects and microenvironmental changes associated with injectable AAM in comparison to conventional fat grafting.

**Methods:**

AAM was manufactured from fresh human abdominoplasty fat using a mechanically modified method and then transformed into an injectable form. Lipoaspirate was harvested employing the Coleman technique. A weight and volume study was conducted on athymic nude mice by injecting either injectable AAM or lipoaspirate into the scalp (*n*=6 per group). After eight weeks, graft retention was assessed through weight measurement and volumetric analysis using micro-computed tomography (micro-CT) scanning. Histological analysis was performed using immunofluorescence staining for perilipin and CD31.

**Results:**

Injectable AAM exhibited similar weight and volume effects in murine models. Histological analysis revealed comparable inflammatory cell presence with minimal capsule formation when compared to conventional fat grafts. Adipogenesis occurred in both AAM-injected and conventional fat graft models, with no significant difference in the blood vessel area (%) between the two.

**Conclusions:**

In summary, injectable AAM demonstrates effectiveness comparable to conventional fat grafting concerning volume effects and tissue regeneration in soft tissue reconstruction. This promising allogeneic injectable holds the potential to serve as a safe and effective “Off-the-Shelf” alternative in both aesthetic and reconstructive clinical practices.

**No Level Assigned:**

This journal requires that authors assign a level of evidence to each submission to which Evidence-Based Medicine rankings are applicable. This excludes Review Articles, Book Reviews, and manuscripts that concern Basic Science, Animal Studies, Cadaver Studies, and Experimental Studies. For a full description of these Evidence-Based Medicine ratings, please refer to the Table of Contents or the online Instructions to Authors www.springer.com/00266.

## Introduction

Autologous fat grafting is considered an ideal technique for soft-tissue augmentation due to its high integration with native tissue and low complication rates [1]. However, a major drawback is the unpredictable volume retention, ranging from 20 to 80%. To address this, numerous studies have refined techniques and protocols [2]. Cell-assisted lipotransfer, a combination of stromal vascular fraction (SVF) or adipose-derived stem cells (ASCs) with conventional fat, has been developed to further enhance the efficacy of fat grafting in breast augmentation [3] and facial rejuvenation [4]. Harvesting a large amount of fat to generate SVF cells is time- and labor-consuming. Moreover, the optimal ratio of supplemented cells to fat remains unclear, and the use of ASCs in clinical settings has not yet been approved due to legal and ethical issues [5]. Therefore, exploring new biocompatible products for soft tissue restoration is essential.

A novel biomaterial isolated from adipose tissue, called acellular adipose matrix (AAM), was the focus of our study. Fat tissue undergoes mechanical, chemical, and biological processes to entirely remove its cellular components [6]. AAM serves as a potential scaffold supporting stem cell proliferation and differentiation, while also inducing adipogenesis and angiogenesis [6, 7]. This product offers minimal immunogenicity [7, 8], making it an off-the-shelf biocompatible filler for plastic and reconstructive surgery and tissue engineering. AAMs of various shapes can be fabricated from human adipose tissue through homogenization, centrifugation, and freeze-drying [9]. Injectable AAM may thus be an appropriate candidate for replacing autologous fat in volume restorations.

This study aims to investigate the volume effect and microenvironmental changes in injectable AAM compared with conventional fat grafting. We hypothesize that AAM can achieve a volume effect, thereby serving as a novel allogeneic grafting method. We believe that our findings will contribute to advancements in soft tissue reconstruction strategies in clinical practice.

## Methods

### Adipose Tissue Harvesting and Injectable AAM Processing

The study protocol received approval from the Institutional Review Board of our institution. Abdominal adipose tissue samples were procured from discarded tissues of patients aged 30–40 undergoing transverse rectus abdominis myocutaneous flap surgery for breast reconstruction. The samples were aspirated and centrifuged at 3000 rpm for 3 min, and conventional lipoaspirate was obtained.

We modified the previous production method for generating fat tissue in AAM through mechanical, chemical, and biological processes [10]. In brief, conical tubes (50 ml) containing adipose tissue were submerged in liquid nitrogen for 10 min and promptly transferred to a 37 °C water bath for 15 min. The freeze-thaw cycle was repeated five times to enhance mechanical properties before centrifugation at 1500*g* for 10 min at room temperature (RT). Following centrifugation, the samples underwent triple washing with phosphate-buffered saline (PBS). After discarding the upper fatty liquid portion, the tissues underwent an 8-h polar solvent extraction using 99.9% isopropanol [11]. Subsequently, the processed tissues were rinsed in PBS four times for 30 min each and incubated in a solution of 0.05% trypsin, 0.05% EDTA, 10 ng/ml DNAse I (Sigma-Aldrich; Merck KGaA, Darmstadt, Germany), and 10 ng/ml RNAse (Sigma-Aldrich; Merck KGaA) for 2 h with slow rotation at 37 °C in an incubator. After four washes with PBS for 30 min each, the tissues were incubated in 1% penicillin (Sigma-Aldrich; Merck KGaA) and streptomycin (Sigma-Aldrich; Merck KGaA) for 12 h at 4 °C and stored in PBS at 4 °C [12]. To convert the manufactured AAM into an injectable form, the final material was finely minced with sharp scissors, air-dried at RT in a UV bench for 4 h, and transferred into a 1cc syringe with an 18G needle.

### Scanning Electron Microscopy (SEM)

The cellular components and matrix architecture of conventional lipoaspirate and AAM samples were examined using SEM. The samples were initially fixed with 2.5% glutaraldehyde for 24 h at 4 °C. Subsequently, they were transferred onto cover glass slides and air-dried at RT. The surface morphology of the AAM was observed using a scanning electron microscope (JSM-7401F) after platinum coating at an accelerating voltage of 15 kV.

### Animal Models and Procedures

Eight-week-old athymic nude mice (weighing 20–25 g) were procured from Koatech (Gyeonggi-do, South Korea) for experimentation. These mice were housed in a temperature-controlled environment at 24 ± 2 °C, maintaining an artificial 12-h light/dark cycle. All applicable institutional and/or national guidelines for the care and use of animals were followed.

A total of 12 mice were subjected to anesthesia using 3% isoflurane in 100% oxygen at a delivery rate of 5 L/min for induction. These mice were randomly allocated into two groups. Subsequently, either lipoaspirate or AAM samples were randomly administered at the supraperiosteal plane of the skull using 18G needles (0.2 ml/sample, *n* = 6 per group).

Eight weeks postoperatively, the grafts underwent evaluation through weight measurements and micro-CT scans using the Quantum GX system (Hopkinton, Mass., USA). Graft dimensions, including height (H), horizontal width (X), and vertical width (Y), were quantified from the micro-CT images utilizing the Caliper Micro-CT Analysis software.

### Histomorphometric Analysis

Tissue samples were fixed overnight in 4% paraformaldehyde (BD Biosciences), dehydrated, and subsequently embedded in paraffin. Slides with a thickness of 4 μm were prepared for hematoxylin-eosin (H&E) and Masson’s trichrome staining. Imaging was performed utilizing a light microscope (Nikon ECLIPSE Ts2; Nikon, Japan).

Unstained slides underwent deparaffinization with xylene, rehydration, and were subjected to antigen retrieval by boiling in citrate buffer (Agilent Technologies, Santa Clara, CA). Following this, samples were blocked with 5% goat serum (Jackson ImmunoResearch, West Grove, PA) for 1 h and subsequently incubated overnight at 4 °C with primary antibodies. After washing, the samples were incubated with secondary antibodies for 2 h at RT. The slides were counterstained with 4',6-diamidino-2-phenylindole (DAPI) to visualize the nuclei. The primary antibodies used were rabbit anti-CD31 (PA5-16301, Invitrogen) and rabbit anti-perilipin (PA5-72921, Invitrogen). A fluorescein-conjugated goat anti-rabbit secondary antibody was employed. Immunofluorescence-stained images were visualized using a confocal microscope (LSM 800; Zeiss, Oberkochen, Germany). Five random fields from each sample were analyzed using ImageJ (Fiji) software (National Institutes of Health, Bethesda, MD).

### Statistical Analysis

The results were analyzed using the nonparametric Mann–Whitney* U* test, chosen due to the small sample size and the non-normal distribution of the data. Statistical analyses were conducted using R Version 4.0.3, and significance was determined at* p*-values < 0.05.

## Results

### Injectable AAM Fabrication

**Fig. 1:**
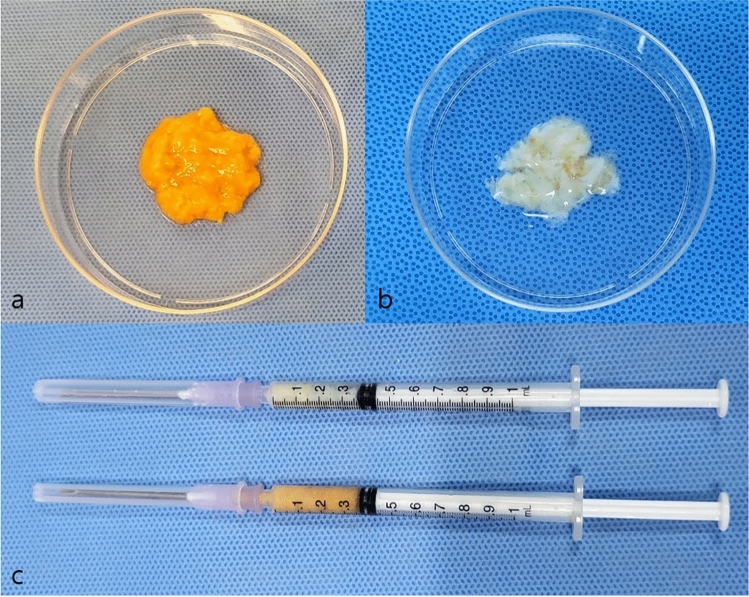
Conventional lipoaspirate by the Coleman technique (**a**) and manufactured AAM (**b**) and both lipoaspirate (downside) and fabricated AAM (upside) prepared in 1cc syringe with 18G needle as injectables (**c**)

Human AAM was produced utilizing a modified protocol developed in our laboratory. The freeze–thaw cycle was limited to five repetitions, as previous studies have demonstrated that more than five cycles do not enhance the effect but may compromise the microstructure of the AAM [13]. The lipoaspirate obtained through the conventional Coleman technique [14] exhibited yellowish, jelly-like features. In contrast, the AAM displayed white, fibrous tissue characteristics. The color change resulted from the repeated washing of the lipid portion, while the fibrous structure was attributed to the collagen-dominant components of the adipose tissue extracellular matrix (ECM) (Fig. 1). After air drying to fabricate the injectable form, the AAM exhibited a transformation, becoming drier, firmer, and finer compared to its original state.

### 3D Microstructure and Properties of AAM

**Fig. 2:**
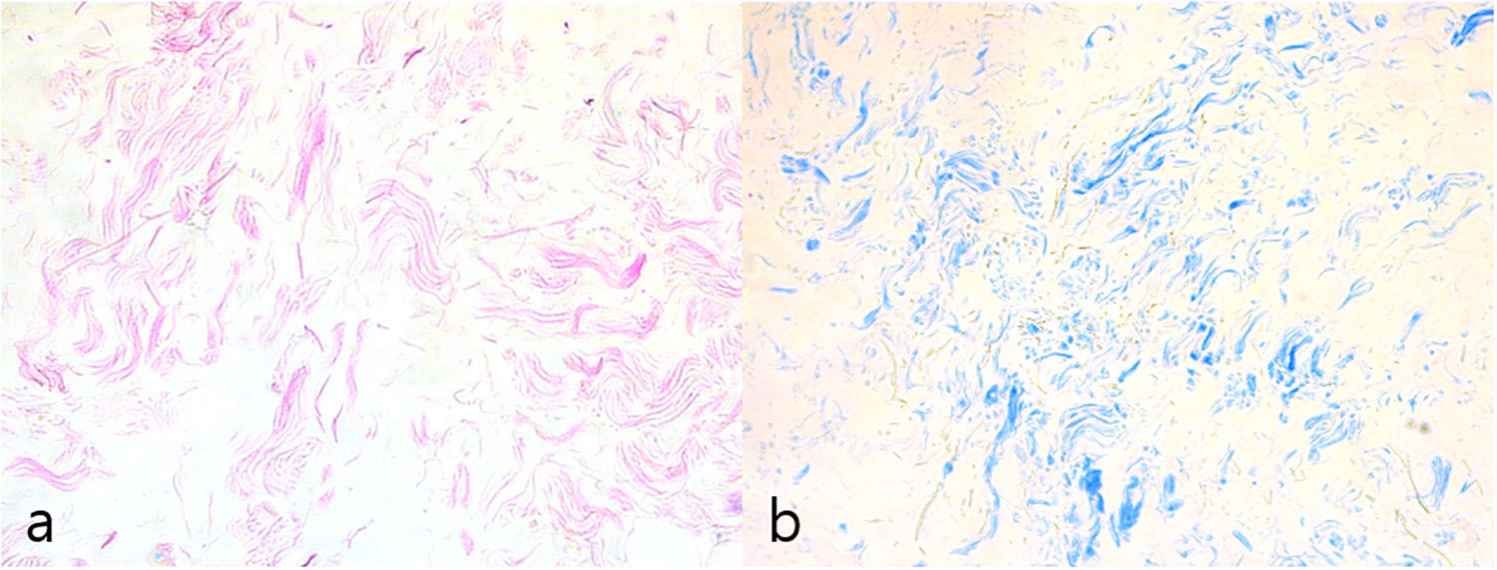
Representative histological H&E stained (**a**) and Masson's trichrome stained (**b**) manufactured human AAM (x100 magnification). H&E staining confirmed the effectiveness of the decellularization protocol, with no residual cells or debris present in the matrix. Masson's trichrome staining to examine the collagen organization in the AAM. The staining confirmed the decellularization protocol's efficacy and identified equally distributed collagen organization

Microscopic examination of SEM images revealed that fresh fat maintained its cellular structure, while the AAM exhibited a finely organized parallel fibrillar structure devoid of cellular components. The SEM image further illustrated that AAM is characterized by an ultrastructure featuring a network-type collagen matrix, accompanied by a prominent basement membrane component in adipose tissue (Fig. 2). This unique structure not only retained the macroscopic 3-D arrangement of collagen fibers but also preserved the organized nano-fibrous collagen 3-D microstructure.

**Fig. 3:**
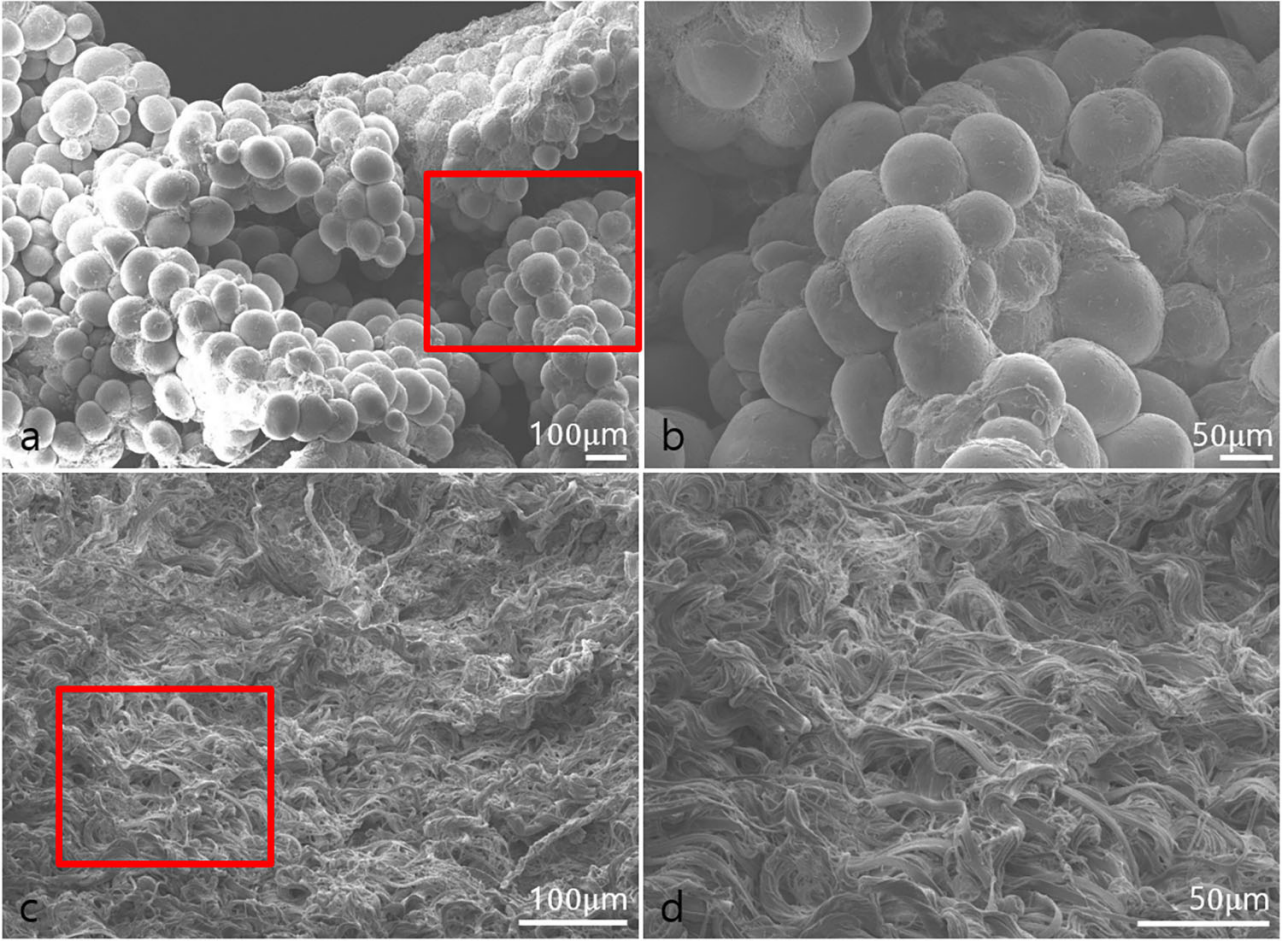
SEM examination of the normal fat tissue (**a, b**) and AAM (**c, d**). The structure of the collagen fibers was evenly distributed in AAM. Scale bar, 200× 100 µm (**a, c**) and 450× 50 µm (**b**,** d**)

The effectiveness of the decellularization protocol was validated through H&E staining of AAM, demonstrating the absence of residual nuclei within the matrix (Fig. 3). To elucidate the composition of AAM, Masson's trichrome staining was employed to visualize the collagen structure, revealing collagen bundles of varying thicknesses. These histological findings affirm the success of our protocol in producing a well-suited acellular adipose matrix.

### Macroscopic Observation of Graft Retention

**Fig. 4:**
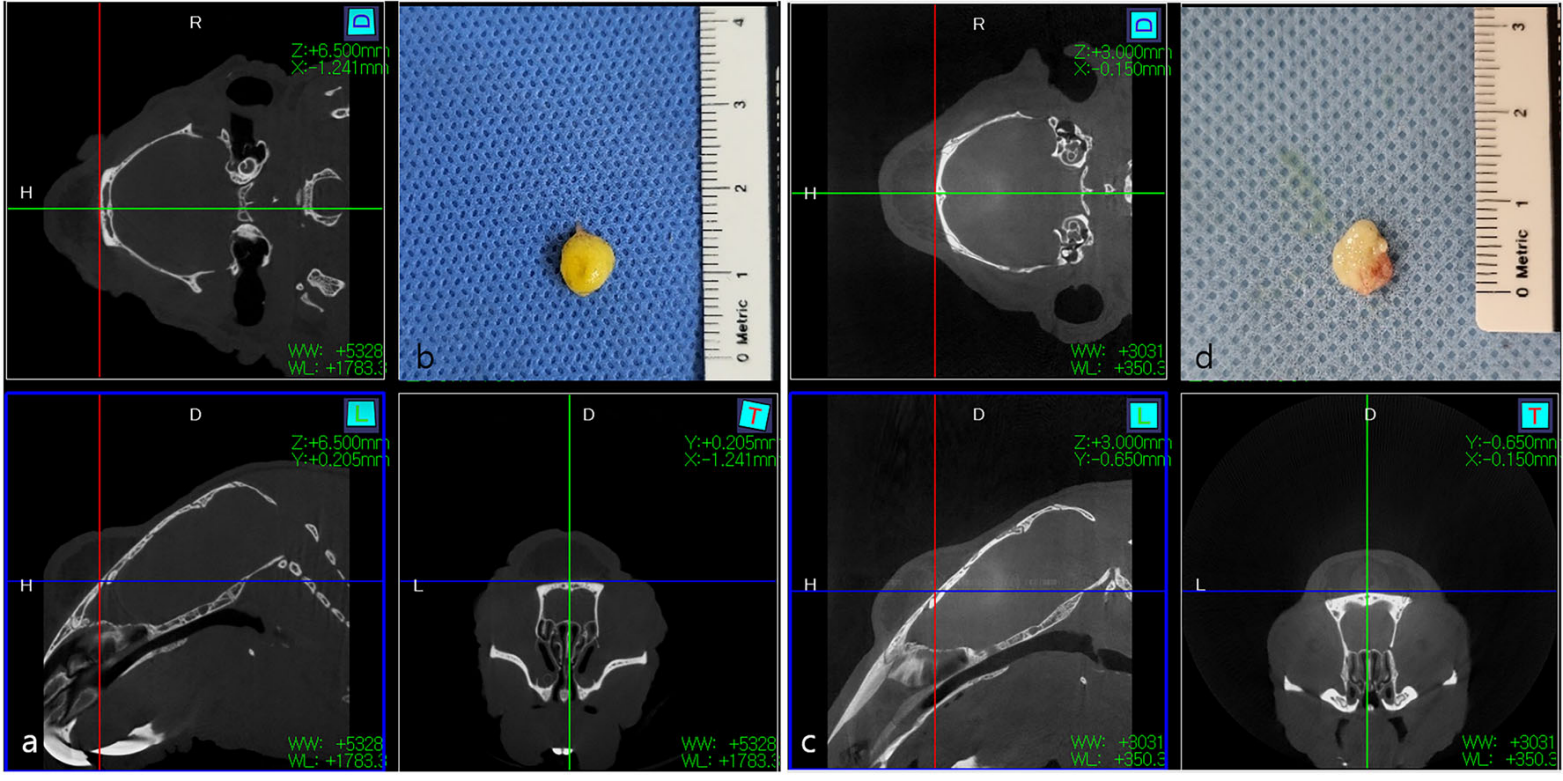
Micro-CT image of injected lipoaspirate (**a**) and AAM (**c**) at 8th week, using Caliper Micro-CT Analysis software. Macroscopic observation of extirpated mass of injected lipoapirate (**b**) and AAM (**d**) at 8th week

At postoperative week 8, the graft portion was assessed using micro-CT with intraperitoneal anesthetic solution infiltration (80 ml/kg alfaxan + 10 mg/kg xylazine). Subsequently, the graft portion was excised using a scalpel and scissors and subjected to macroscopic photography (Fig. 4). Following macroscopic observation of each injected AAM and lipoaspirate, five out of six samples were randomly chosen and fixed overnight in 4% paraformaldehyde (BD Biosciences) for subsequent histological analysis.

In the injectable AAM group, the grafted mass exhibited a mixture of yellowish and whitish colors, while the lipoaspirate group displayed a pure yellowish color. The masses were round but slightly flatter in the injectable AAM group. Both sets of masses were firm and mobile, with those in the injectable AAM group being slightly more elastic and softer.

Neovascularization was evident in the grafted masses of the injectable AAM group, highlighting the angiogenic effects of injectable AAM materials.

The graft height (H(mm)), horizontal width (X(mm)), and vertical width (Y(mm)) were measured on micro-CT images using Caliper Micro-CT Analysis software (Fig. 4). The height (H) of the Injectable AAM group was lower than that of the lipoaspirate group, but the difference was not statistically significant (Injectable AAM group: median = 2.3mm, interquartile range = 1.9–2.0 mm; Lipoaspirate group: median = 2.5 mm, interquartile range = 2.4–2.8 mm, *p* = 0.2257, *n* = 6). The horizontal width (X) was higher in the Injectable AAM group, though not statistically significant (Injectable AAM group: median = 8.7 mm, interquartile range = 8.6–9.6 mm; Lipoaspirate group: median = 8.4mm, interquartile range = 8.1–9.0 mm, *p* = 0.5196, *n* = 6). The vertical width (Y) was also higher in the Injectable AAM group, without statistical significance (Injectable AAM group: median = 7.6mm, interquartile range = 7.1–8.1 mm; Lipoaspirate group: median = 6.6mm, interquartile range = 6.3–7.1 mm, *p* = 0.0651, *n* = 6). These findings indicate that the height (H), horizontal width (X), and vertical width (Y) of the injected materials did not significantly differ between the two groups.

### Quantitative Analysis of Graft Retention

**Fig. 5:**
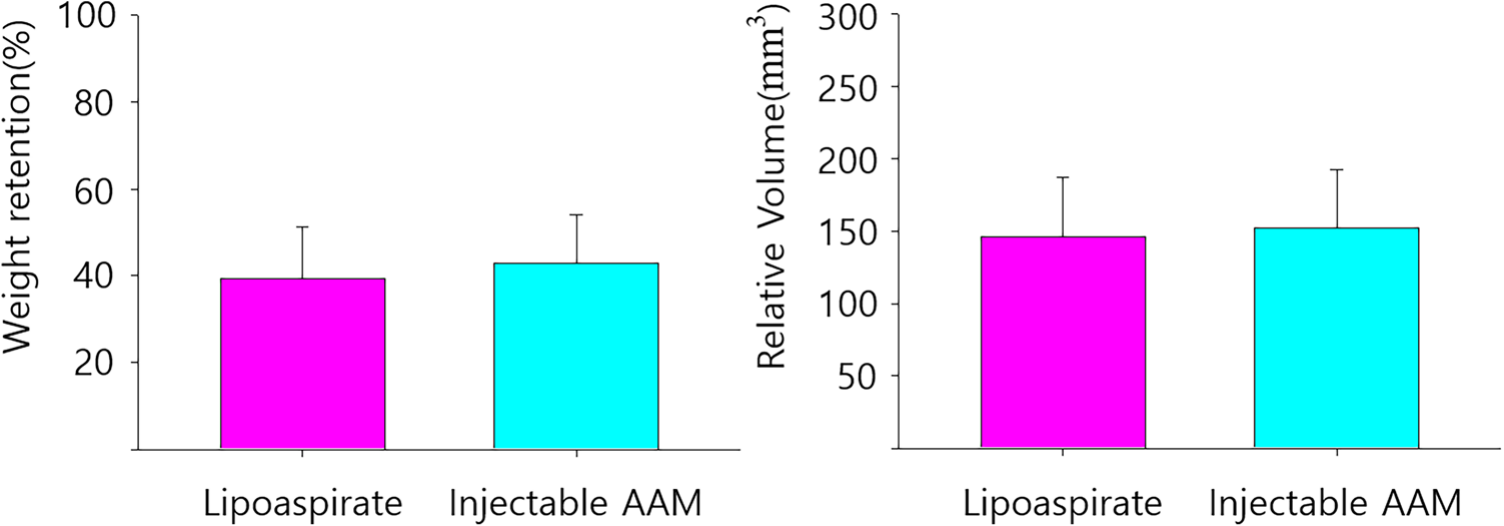
The average weight retention (%) (left) and relative volume (mm^3^) (right) of the injected material were statistically not different between the lipoaspirate group and the injectable AAM group. The Mann–Whitney *U* test was used for analysis; data were presented as the mean ± standard deviation; *n* = 6 per group

The initial weights of 0.2 mL injectable AAM and lipoaspirate were measured at week 0. The mean weights from the three measurements of 0.2 ml injectables were 0.175 g for AAM and 0.165 g for lipoaspirate. The final weights of the grafted materials were measured after extirpation at week 8, and the weight retention (%) was calculated for each sample by dividing the final weight by the initial weight of the material. The weight retention (%) of the injected materials did not show any statistically significant difference between the Injectable AAM and lipoaspirate (Injectable AAM group: median = 44.857%, interquartile range = 35.429–49.143%; lipoaspirate group: median = 41.818%, interquartile range = 36.212–46.515%, *p* = 0.9362, *n* = 6) (Fig. 5).

To compare the volume retention ratio between the injectable AAM group and the lipoaspirate group, the value of [(height, H(mm)) × (horizontal width, X(mm)) × (vertical width, Y(mm))] was calculated and represented as the estimated final relative volume. The initial volume was 0.2 mL in both groups. Using these calculated values, we compared the estimated volume retention ratios between groups. The relative volume retention of the grafted materials did not exhibit any statistically significant difference between the Injectable AAM and lipoaspirate groups (Injectable AAM group: median = 142 mm^3^, interquartile range = 124–179 mm^3^; lipoaspirate group: median = 151 mm^3^, interquartile range = 138–165 mm^3^, *p* = 1, *n* = 6) (Fig. 5).

### Histologic Findings of Graft Retention

**Fig. 6:**
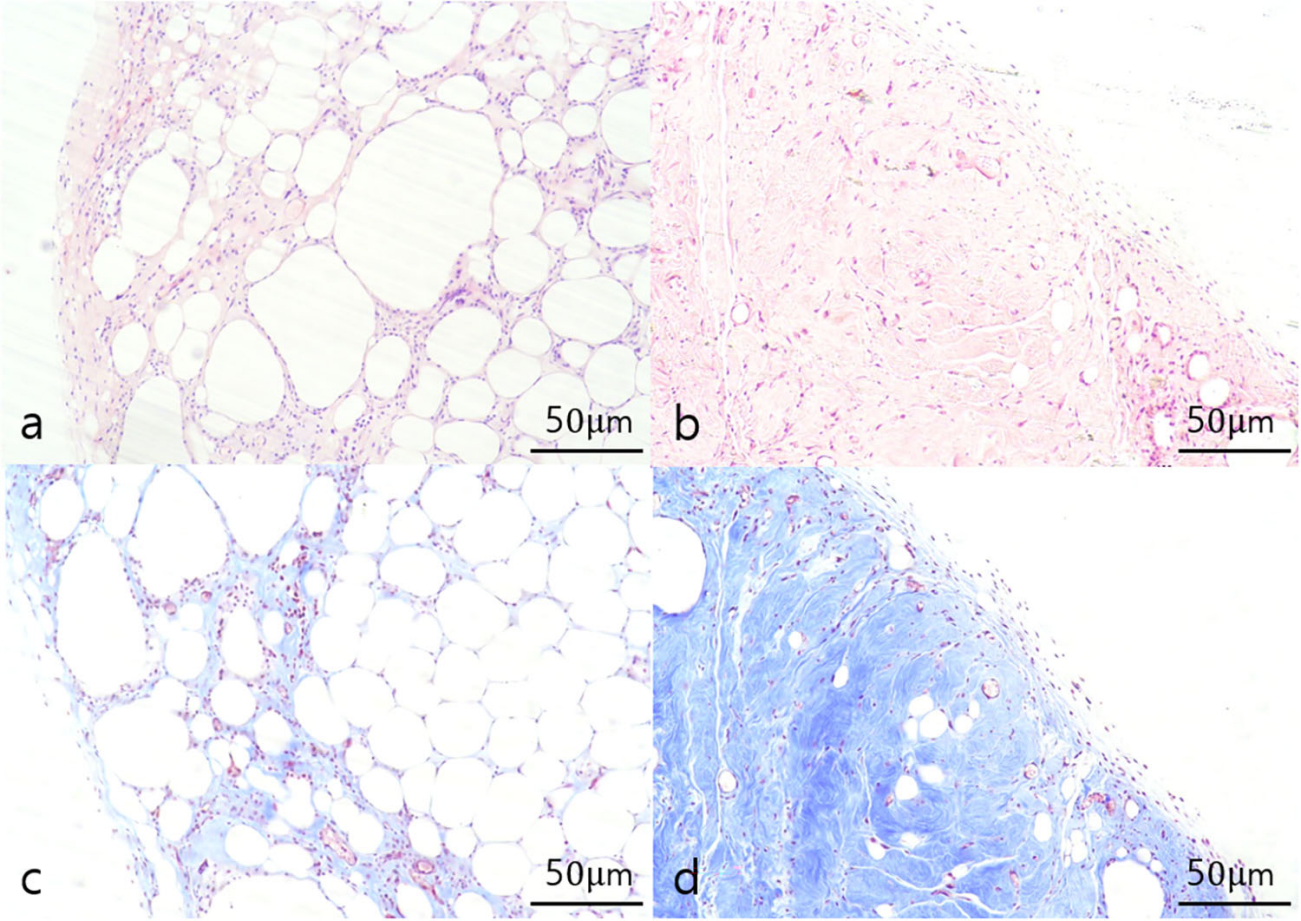
Histologic findings of a section of extirpated mass of lipoaspirate group (**a, c**) and injectable AAM group (**b, d**). Adipocytes and inflammatory cell infiltrations are observed in H&E sections (**a, b**) and collagenous fibrous tissue component and peri-mass capsule are observed in Masson’s Trichrome sections (**c, d**). Magnification 200×, scale bar, 50 µm

For histological analysis, five randomly selected graft samples were extracted from each group, comprising a total of six groups. H&E staining was employed to identify adipocytes and inflammatory cells, while Masson's trichrome staining was utilized to delineate collagen distribution and peri-graft encapsulation. In the lipoaspirate group, adipocyte-dominant fields were prominent, contrasting with predominantly collagenous fibrous tissue-dominant fields in other groups (Fig. 6). To assess the degree of inflammation in each slide, a five-point scale was employed (grade 0: none, grade 1: 0–20%, grade 2: 20–40%, grade 3: 40–60%, grade 4: 60–80%, grade 5: 80–100% of inflammatory cells shown in the slides), evaluated independently by two authors. Inflammatory cell infiltration in the grafted materials showed no statistically significant difference between the Injectable AAM and lipoaspirate groups (Injectable AAM group: median = 1, interquartile range = 1 to 2; lipoaspirate group: median = 2, interquartile range = 2–2, *p* = 0.5637, *n* = 5).

The thickness of the peri-graft capsule was assessed by randomly measuring three points of the capsule on each slide and calculating the average capsule thickness. The results indicated no statistically significant difference in peri-graft capsule thickness (μm) when comparing the Injectable AAM group and the lipoaspirate group (Injectable AAM group: median = 13.6 μm, interquartile range = 12.5–18.3 μm; Lipoaspirate group: median = 19.3 μm, interquartile range = 12.3–22.5 μm, *p* = 0.5309, *n* = 5).

### Analysis of Adipogenesis and Angiogenesis

**Fig. 7:**
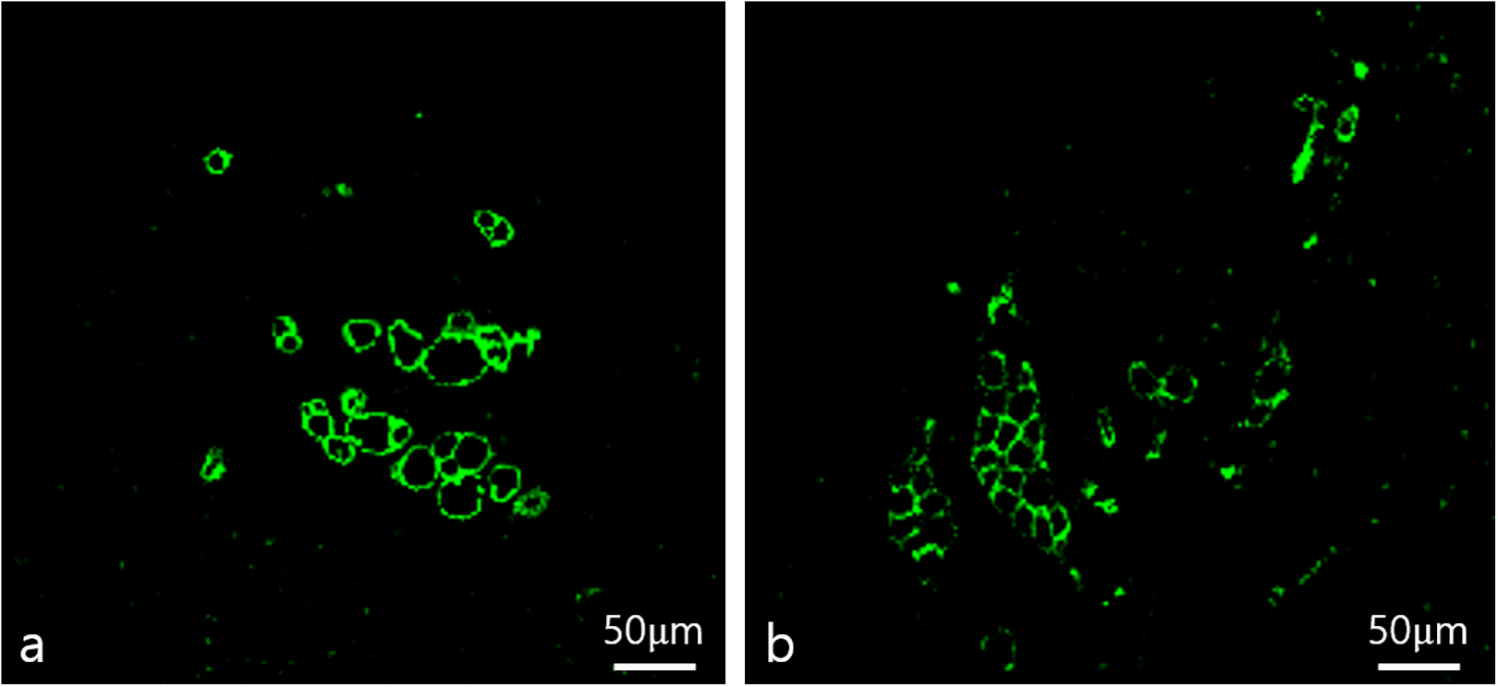
Immunofluorescent staining of adipocytes of a section of extirpated mass of lipoaspirate group (**a**) and injectable AAM group (**b**). The green signals refer to perilipin (+) adipocytes, which imply adipogenesis. Scale bar, 50 µm

**Fig. 8:**
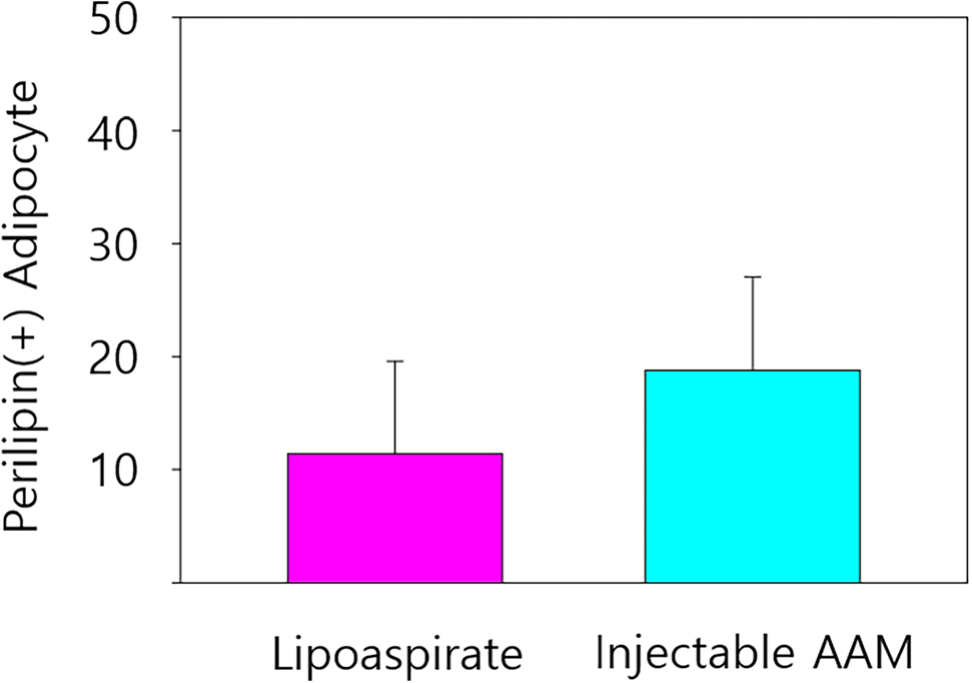
The perilipin (+) adipocyte count in the injectable AAM group was significantly not different compared to that of the lipoaspirate group. The Mann–Whitney *U* test was used for analysis; data were presented as the mean ± standard deviation; *n* = 5 per group

**Fig. 9:**
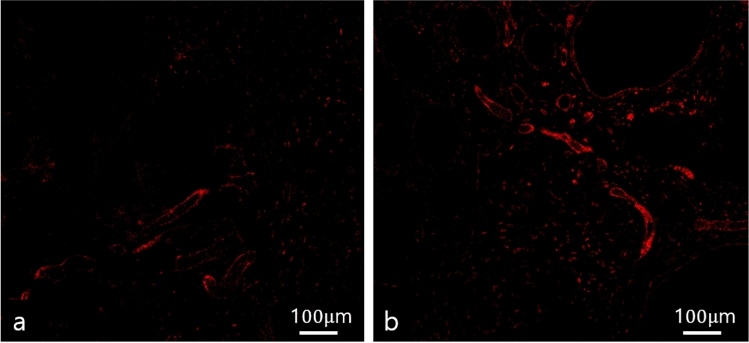
Immunofluorescent staining of endothelial cells of a section of extirpated mass of lipoaspirate group (**a**) and injectable AAM group (**b**). The red signals refer to CD 31 (+) cells, which imply angiogenesis. Scale bar, 100 µm

**Fig. 10:**
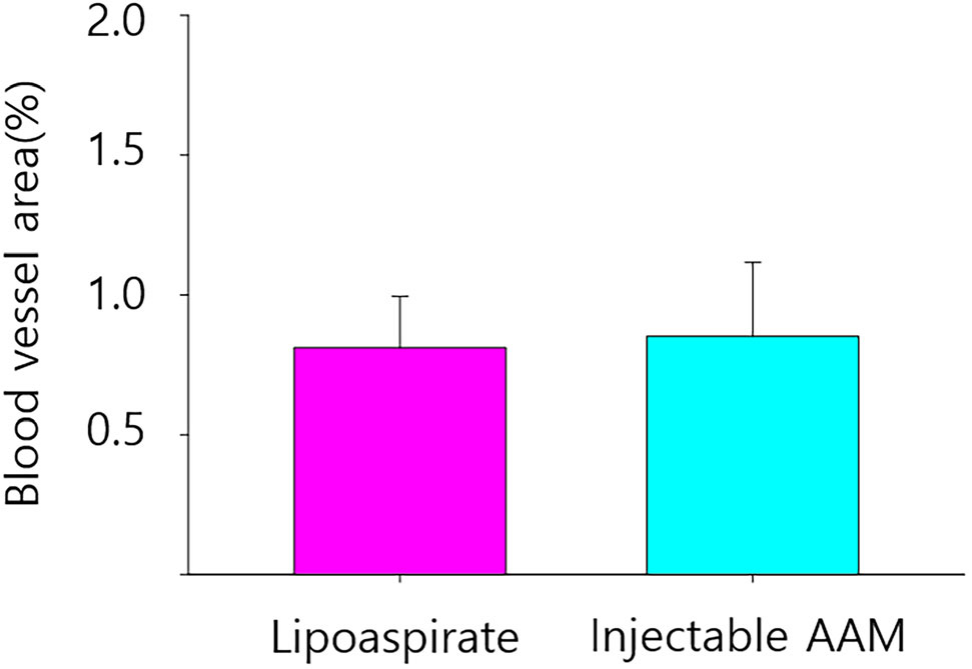
The blood vessel area (%) in the injectable AAM group was significantly not different compared to that of the lipoaspirate group. The Mann–Whitney *U* test was used for analysis; data were presented as the mean ± standard deviation; *n* = 5 per group

Immunofluorescence staining was performed on five paraffin block samples from both groups, and the samples were visualized using a confocal microscope (LSM 800; Zeiss, Oberkochen, Germany). Perilipin staining was employed to detect adipogenesis in the graft materials, while CD31 staining served to identify angiogenesis, a marker of endothelial cells in blood vessels. Many perilipin-positive cells were observed in the injectable AAM group, indicating adipogenesis occurred in the AAM material without live donor adipocytes. As a control group, numerous perilipin-positive adipocytes were also found in the lipoaspirate group, which is well-known for conventional fat grafting (Fig. 7). For a quantitative evaluation of adipogenesis, five 500 μm × 500 μm-sized fields were randomly selected, and perilipin-positive adipocyte counts were measured. The average count of each slide was analyzed, and there was no significant difference in perilipin-positive adipocyte number between the injectable AAM group and the lipoaspirate group (Injectable AAM group: median = 15, interquartile range = 12–25; Lipoaspirate group: median = 8, interquartile range = 6–13, *p* = 0.1719, *n* = 5) (Fig. 8). CD31 positive signals were detected, indicating angiogenesis within the injected materials. These signals were widely distributed in both the injectable AAM and lipoaspirate groups (Fig. 9). For a quantitative evaluation of angiogenesis, five 500 μm × 500 μm-sized fields were randomly selected, and %Area values were measured using ImageJ (Fiji) software. This value represents the blood vessel area (%), and the average value of each slide was collected and analyzed. There was no significant difference in CD31-positive cells between the injectable AAM group and the lipoaspirate group (Injectable AAM group: median = 0.804%, interquartile range = 0.708–0.938%; Lipoaspirate group: median = 0.787%, interquartile range = 0.756–0.838%, *p* = 1, *n* = 5) (Fig. 10).

## Discussion

The concept of AAM did not emerge recently. Decellularization of specific tissues to supplement tissue regeneration has been a steadfast focus in tissue engineering for decades. The unique biological and physical characteristics of the ECM play a crucial role in determining stem cell behavior. With the rapid advancement of adipose tissue engineering, AAM has garnered attention due to its broad range of sources and superior regeneration capacity compared to other specific tissues such as the skin, heart, cartilage, and lungs [13]. In 2010, Flynn et al. pioneered comprehensive methods for the decellularization of adipose tissue. They successfully achieved complete decellularization of adipose tissue samples weighing up to 25 g, which, upon hydration, typically represented 30–45% of the original tissue mass [11]. Subsequently, numerous researchers have adopted and adapted this protocol with minor variations to further refine the concept of adipose tissue decellularization.

Several research groups have initiated investigations into the utilization of AAM as a scaffold for tissue engineering. This approach exhibits considerable promise as a carrier for delivering adipose stem cells and as a construct that facilitates soft-tissue regeneration [15]. Brown et al. explored the potential of AAM as a scaffold for enhancing adipogenesis through adipose stem cell supplementation [16]. Young and Christman delved into the examination of commercially available soft-tissue fillers, synthetic and natural polymers, with and without ECM-based materials, for adipose tissue engineering. Their findings indicated that ECM-based products hold the highest potential for promoting de novo adipogenesis and, consequently, facilitating long-term retention [17].

Although there have been many studies on the role and effect of AAM, few have been conducted on the volume effect in soft tissue reconstruction from a clinical perspective in plastic and reconstructive surgery. To the best of our knowledge, this is the first study to compare the volume effect with microenvironmental changes in soft tissue reconstruction with conventional fat grafting, which is currently the standard, safe, and effective method for treating soft tissue defects and contour abnormalities. The purpose of our study was to prove the effectiveness of substances that have not yet been clinically approved for use in preclinical studies and to accumulate data to link them to future clinical studies after approval.

The volume effect includes not only volume retention, but also the effect of maintaining the initial contour, texture, and augmented height or length. Unlike previous studies that examined the volume retention of AAM, this study sought to comprehensively analyze the overall effects from a clinical surgeon’s perspective.

In addition, we fabricated AAM in an ‘injectable form’, which is contained in a 1cc syringe and can be used in the same way as fat grafts or fillers. Although there are previous basic studies on transforming an initially manufactured cross-linked mass, such as AAM, into power- or gel-type materials [18], there is a lack of preclinical volume effect studies dealing with injecting AAM in an injectable form in a clinical setting. More recently, Anderson et al. [19] examined an immunologically active, adipose-derived extracellular matrix biomaterial for soft tissue reconstruction in vitro and in vivo; however, AAM was implanted after incision in experimental mice, which is a surgical procedure rather than a simple outpatient-based injection procedure in the clinical field. Thus, as this study aimed to prove its effectiveness by comparing the clinical use of AAM with that of existing fat grafting in the same environment, the results of volumetric and histological analyses could be much more meaningful.

In this animal experimental model, the follow-up period was two months. This period has been commonly used in similar previous studies in murine models with fat grafting, so there will not be much controversy. However, although fat grafting has been established for a long time, many studies are still ongoing, and new facts are being revealed about the fate of adipocytes and ASCs in the microscopic scope, as well as volume changes and cell replacements, over time after fat grafting are available. In relation to this, if additional research is conducted on AAM interacting with stem cells, the volume effect, and fate over time, it will add to the breadth and depth of the current adipose tissue-related research trends.

Our study findings demonstrated that the volume effect of AAM is similar to that of clinically well-known conventional fat grafting. Furthermore, AAM has potential of clinical benefit because of its convenience. This ready-made product is easier to be used for patients than lipoaspirate which should be harvested from donor sites of the patients before injection procedures. Also, this could save the patients’ time and get rid of donor site morbidity. Thus, even though there might be a financial burden to use AAM as a “Off-the-Shelf” merchandise, it is still an attractive and competitive material from a cost-effective point of view. This material could reduce not only time, but also physical and psychological strain of the patients due to its unique advantages and convenience.

From a microscopic perspective, we examined the representative microenvironment of adipogenesis and angiogenesis using antibodies against perilipin and CD 31 using confocal microscopy. First, we confirmed the occurrence of adipogenesis. Although the theoretical background has already been revealed, it is noteworthy that this occurred in this preclinical experimental model. The experiment was conducted under the same conditions as conventional fat grafting and adipogenesis originating from the host was observed through the injection of acellular material, suggesting that a similar reaction is likely to occur in clinical practice with the same material. In addition, in the quantitative analysis of angiogenesis, the fact that neovascularization occurs when the injection is performed, similar to fat grafting in this experimental model, is noteworthy in that it is a result obtained together with this volume effect study, although this fact has already been proven in other in vitro or in vivo study designs.

This study has some limitations. In terms of study scale, the study included only a small number of mice in each group, lacked heterogeneity of donor fat, examined a one-point observation period, and lacked long-term follow-up. In terms of the study design, the study did not include the diversity of graft retention depending on the injected volume or storage period of injectable AAM before use. The final volume was not measured directly. Further studies are required to overcome these limitations.

## Conclusion

Injectable AAM demonstrates effectiveness comparable to conventional fat grafting in terms of volume and tissue regeneration in plastic and reconstructive surgery. This promising allogenic injectable holds the potential to provide a safe and effective 'Off-the-Shelf' alternative for soft tissue reconstruction in both aesthetic and reconstructive aspects of clinical practice.
